# Improved Metabolic Control in Diabetes, HSP60, and Proinflammatory Mediators

**DOI:** 10.1155/2012/346501

**Published:** 2012-08-12

**Authors:** Claudio Blasi, Eunjung Kim, Anne A. Knowlton

**Affiliations:** ^1^Centro Diabete, ASL RMB-1D, L.go T. Solera n.7, Rome, Italy; ^2^St. Mary's Hospital of Daejeon Catholic University, Clinical Research Institute, Daeheung-dong, Daejeon, Republic of Korea; ^3^Cardiovascular Division, Department of Medicine and Department of Medical Pharmacology, UC Davis Health Systems, Sacramento, CA 95817, USA

## Abstract

The diabetes-atherosclerosis relationship remains to be fully defined. Repeated prolonged hyperglycemia, increased ROS production and endothelial dysfunction are important factors. One theory is that increased blood levels of heat shock protein (HSP)60 are proinflammatory, through activation of innate immunity, and contribute to the progression of vascular disease. It was hypothesized that improvement of diabetes control in patients presenting with metabolic syndrome would lower HSP60, and anti-HSP60 antibody levels and decrease inflammatory markers. Paired sera of 17 Italian patients, before and after intensive treatment, were assayed for cytokines, HSP60 and anti-HSP60 antibodies. As expected, intensive treatment was associated with a decrease in HgbA1C (*P* < 0.001) and BMI (*P* < 0.001). After treatment, there was a significant decrease in IL-6 (*P* < 0.05). HSP60 levels were before treatment −6.9 + 1.9, after treatment −7.1 + 2.0 ng/mL (*P* = ns). Overall HSP60 concentrations were lower than published reports. Anti-HSP60 antibody titers were high and did not decrease with treatment. In conclusion, improvement of diabetic control did not alter HSP60 concentrations or antiHSP60 antibody titers, but led to a reduction of IL-6 levels.

## 1. Introduction

 Atherosclerosis is more widespread and severe in diabetics [1]. It is characterized by a greater inflammatory involvement, exposing patients to a high risk of cardiovascular (CV) events [2, 3]. Myocardial infarction is 2 to 4 times more frequent, and increased cardiovascular risk remains even after controlling for other concomitant factors like hypertension and dyslipidemia. Diabetics' cardiovascular mortality exceeds 70%. Epidemiological studies demonstrated that even a small reduction of glycosylated hemoglobin corresponds to a reduction of CV risk, but tight control has been associated with no reduction in CV mortality [4–6].

 Despite a great deal of research, the cellular and molecular mechanisms underlying the glucose-atherosclerosis relationship are not fully understood. There are multiple potential pathways to endothelial injury and the vascular complications of diabetes, including chronic inflammation, increased oxidative stress, and activation of the immune response—both innate and adaptive [3, 7, 8]. Once the initial dysfunction in the endothelium occurs, chronic inflammation and an immune response contribute to the progression of vascular disease. Autoimmunity is one mechanism of vascular injury in diabetes. A key-identified antigen is heat shock protein (HSP)60, a protein that has been found on the surface of stressed endothelial cells [9, 10]. Anti-HSP60 antibodies have been found in the serum of many individuals, and they are thought to increase in a number of disease states. HSP60 is also an important endogenous inflammatory mediator. Toll-like receptors (TLR), part of the innate immune response, are present on the endothelial cell membranes and recognize HSP60 present in the circulation. The binding of HSP60 to endothelial cell TLRs will result in the activation of NF*κ*B, leading to increased expression of inflammatory genes and the release of pro-inflammatory cytokines, including TNF*α* and IL-6.

We hypothesized that in diabetes there would be a decrease in serum HSP60, HSP60 antibodies and in inflammatory cytokines with improved glycemic control. We investigated the effect of good metabolic control on serum HSP60, HSP60 antibodies, and inflammatory cytokines in type-2 diabetic patients to evaluate the influence of hyperglycemia on autoimmune and inflammatory indicators.

## 2. Methods

### 2.1. Patient Data

Diabetic patients from one of our clinics (CB) were enrolled in a study to determine the effect of glycemic control on inflammatory endpoints and the release of HSP60 into the serum. Paired sera were collected from 17 diabetic patients (10 women and 7 men, mean age 62.3 ± 2.1 years), before and after having intensive treatment for glycemic control. Local committee approval was obtained and patients gave informed consent. Subject characteristics are shown in Table 1. Samples were stored at −80°C until use. 

*HOMA (homeostasis model assessment, *an index of insulin-resistance) was calculated based on the following formula: (blood glucose [mmol/L] × insulin level [*μ*U/mL])/22.5.

*Glycosylated hemoglobin(HgbA1C)* was measured by the hospital clinical laboratory. The routine assay involves an automated analytical system based on a cation-exchange HPLC method. The procedure is the reference DCCT method.

### 2.2. Serum HSP60

A commercial ELISA (Assay Designs) was used to measure serum HSP60 levels. Samples were diluted 1 : 20 before analysis based on pilot studies.

### 2.3. Serum Anti-HSP60

*Slot-Blots*—a standard method in immunology, were used to assess anti-HSP60 antibody titers. This method is distinct from the slot blot method, which is a rectangular version of dot blots. We used an apparatus manufactured by BioRad, which provides 20 vertical slots, designed for testing small serum samples for antibodies. The apparatus is the size of a small SDS PAGE gel. We ran a set of molecular weight markers and a gel-wide band of recombinant human HSP60 (StressGen) on gel, and then transferred to nitrocellulose. The membrane was stained with Ponceau Red (Sigma) to verify the presence of the single *HSP60 protein band across the width of the membrane. A copy of the membrane was made to allow comparison of the final film with the distribution of protein on the membrane*. The prestained molecular weight markers (New England Biolabs) also provided another reference point. We then placed the membrane in the slot-blotting apparatus, and added diluted serum samples based on the range seen in pilot studies. The slot blot is incubated in the apparatus on a rocker panel so that the sample goes back and forth in its long, vertical slot the height of the blot. After incubation, the sample is aspirated out, and the slots washed several times. Then the blot is removed from the apparatus and incubated with anti-human IgG-peroxidase as with a standard western. The end result after development is tiny bands which one can test for aligning with the blot wide band of HSP60. It is not uncommon to see non specific bands at other sizes, which would be read as a positive in a 96 well plate.

This device has 20 vertical slots that permit loading of 20 different serum samples to develop the membrane. Samples were diluted 1 : 100 and 1 : 250 in PBS (phosphate-buffered saline) based on pilot studies. Following incubation for 1 hour on an orbit shaker, serum was aspirated out of the device, the slots washed with TBST, and the membrane removed. The membrane was incubated with anti-human IgG-HRP (Amersham) at 1 : 1000. The blot was then developed with ECL (Pierce) as previously described [11]. The 20 blot lanes were then each interpreted as positive or negative based on the presence or absence of a band matching the location of the recombinant human HSP60 on the membrane. In contrast to dot blotting for antibody titers, this approach confirms the *specificity* of a positive result.

### 2.4. Cytokine Assay

An inflammatory human cytokine cytometric bead array (BD Biosciences) was used to measure IL-1*β*, IL-6, IL-8, IL-10, IL-12 and TNF*α* following the directions of the manufacturer.

### 2.5. Statistics

Data is reported as mean values ± the standard error of the mean (SEM). A *P* < 0.05 was considered to be significant. Paired data from before treatment to optimized treatment was compared using a paired *T* test or Wilcoxon Signed Rank test, where indicated. Multi-variate analysis was performed by Pearson Correlation (SigmaStat). A *P* < 0.05 was considered to be significant.

## 3. Results 

### 3.1. Baseline Diabetes Indices

**Figure 1:**
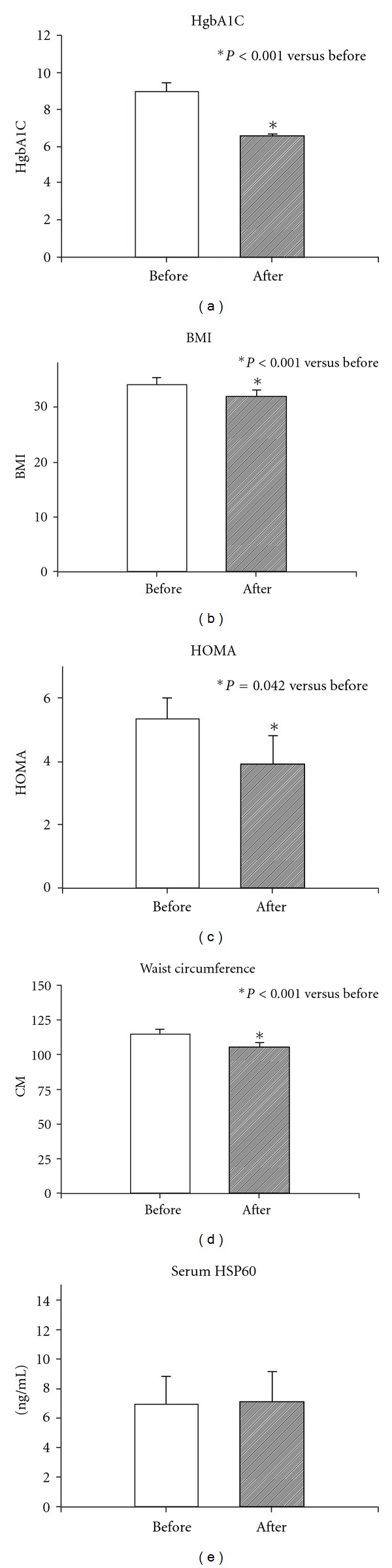
(a) HgbA1C before and after intensive treatment. (b) BMI before and after intensive treatment. (c) HOMA before and after intensive treatment. (d) Waist circumference before and after intensive treatment. (e) Serum HSP60 levels by ELISA before and after intensive treatment. *P < 0.001 except c, where *P* < 0.05.

HgbA1C demonstrated a significant drop with optimized treatment, as expected (*P* < 0.001, Figure 1(a)). Significant reductions of BMI (*P* < 0.001, Figure 1(b)), HOMA (*P* < 0.001) (Figure 1(c)), and waist circumference (*P* < 0.001, Figure 1(d)) occurred, demonstrating significantly improved control of diabetes.

### 3.2. HSP60

HSP60 was present in the serum of both before and after treatment patients (Figure 1(e)). Absolute HSP60 levels before treatment were −6.9 ± 1.9, after −7.1 ± 2.0 ng/mL (*P* = ns). Three patients had no HSP60 in their sera either before or after treatment. There was no correlation between diabetes duration and HSP60 levels, nor was there a correlation with the medication(s) used for diabetes treatment. 

### 3.3. Anti-HSP60 Antibodies

**Figure 2:**
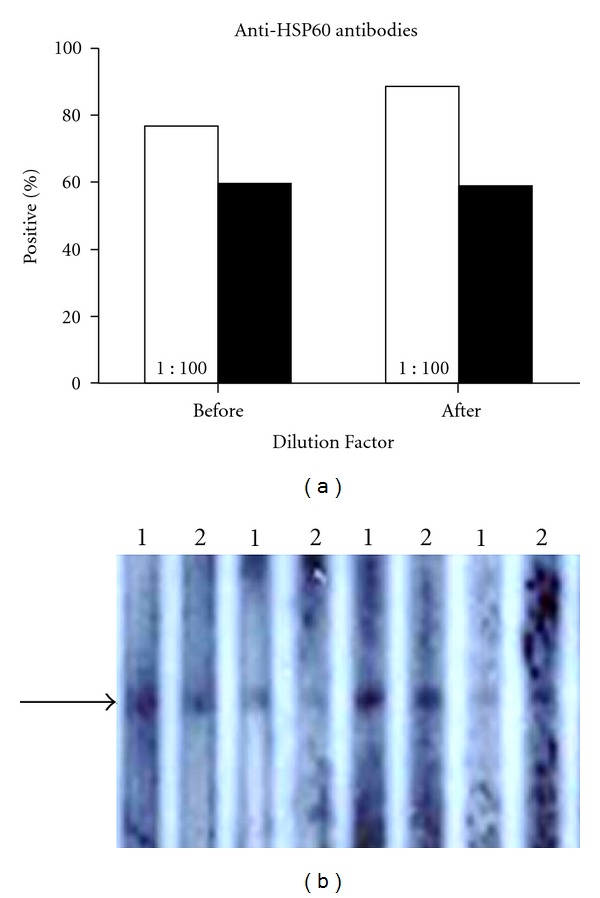
(a) Graph summarizes anti-HSP60 antibody titers before and after intensive treatment. Antibody levels were tested at two different dilutions: 1 : 100 (white bars) and 1 : 250 (black bars). (b) Representative slot blot. Arrow marks location of HSP60 on membrane. Lanes were read as positive or negative at a given dilution based on the presence of a band at the level of the arrow, which marks the location of the recombinant human HSP60 on the membrane. 1— 1 : 100; 2—1 : 250.

Anti-HSP60 antibodies were detected by slot blotting. The graph summarizes the results of 1 : 100 and 1 : 250 dilutions of plasma (Figure 2(a)). With a 1 : 100 dilution, anti-HSP60 antibodies were present in 76.5% before, and 88.2% after (*P* = ns). This decreased to 58.8 and 58.8% at 1 : 250. A representative slot blot is shown in Figure 2(b). 

### 3.4. Cytokine Levels

**Figure 3:**
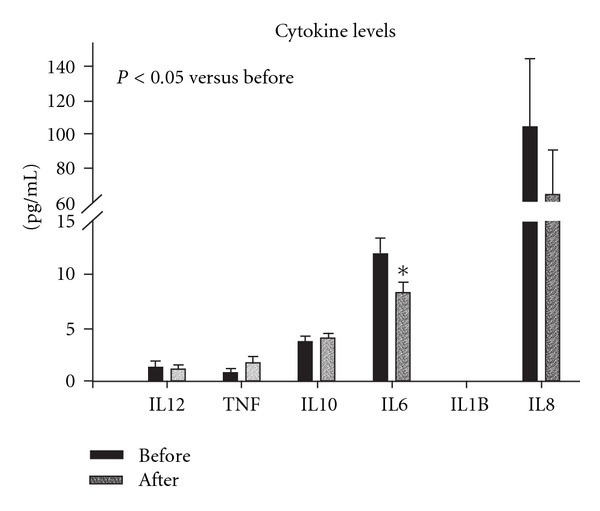
Multiple inflammatory cytokines were measured by flow cytometry using a cytometric bead array. Graph summarizes results. Black bar is before intensive treatment and grey bar after intensive treatment. No IL-1*β* was detected in any samples. *P < 0.05 versus before treatment.

As shown in Figure 3, IL-6, IL-8, IL-10, IL-12 and TNF*α* were detected in the serum both before and after intensive treatment. IL-1*β* was undetectable. Only IL-6 showed a significant reduction with treatment (*P* < 0.05). Overall, IL-8 levels dropped, but there was considerable variation in levels and therefore the change did not reach significance.

### 3.5. Correlation Analysis

A Pearson product moment correlation analysis was performed to identify correlation amongst the clinical findings and inflammatory markers analyzed. As shown in Table 2, before treatment, there was a negative correlation between HSP60 and IL-12. There were borderline correlations between HSP60 and IL-10 and IL-6. As would be expected, there was a positive correlation between BMI and waist circumference. After intensive treatment, again a correlation between BMI and waist circumference was found, and in addition a negative correlation between HOMA and IL-10 was also found. TNF*α* levels showed a correlation with IL-12 levels (*P* = 0.023). There was a borderline correlation of HSP60 and TNF*α* levels, but this did not reach significance (*P* = 0.055). In the present study, leptin and adiponectin were not tested because, unlike cytokines, they are not considered markers of inflammation and are predominantly of adipocytic origin. 

## 4. Discussion

 The underlying mechanisms for the far greater severity of vascular disease in diabetics remain to be fully defined [3, 8]. Endothelial dysfunction, characterized by increased adhesion molecules, increased proinflammatory markers, increased pro-thrombotic factors, increased ROS and loss of normal regulation of vascular tone, occurs in diabetes, likely a result of the convergence of multiple proinflammatory mechanisms [2]. Endothelial dysfunction is accompanied by the development of an autoimmune reaction that appears to play an important role in the inflammatory evolution of atherosclerosis [9, 12]. There are a number of molecules, which are thought to contribute to this process, including oxidized(ox) LDL, advanced glycation end-products, and HSP 60, with oxLDL and HSP60 acting as autoantigens [9]. The oxLDL likely are a result of ROS action on LDL, which have penetrated the subendothelial space. Activation of the immune system through chronic infection or exposure of intracellular proteins, such as HSP60, which are then recognized as danger associated molecular patterns (DAMPs), can lead to inflammation and cell apoptosis and contribute to the development and progression of vascular disease [3, 13]. 

 HSP60 is primarily a mitochondrial protein, where it is critical for proper folding of key metabolic proteins, but HSP60 is also found in the cytosol, where in the heart it has an antiapoptotic role [14]. Diabetes has been reported to be associated with very high levels of HSP60 in blood [15]. HSP60 associates with the cell membrane under stress conditions, and in heart failure localized to the surface of cardiac myocytes, which correlated with myocyte apoptosis [10, 16]. HSP60 has been identified as a potential ligand for TLR4. We have recently shown that TNF*α* and HSP60 each drives the expression of the other, which could explain the correlation between HSP60 and TNF*α* levels [17]. HSP60 has been shown to activate endothelium, smooth muscle cells, and macrophages [18]. TLR4 activation and expression increase in diabetes in response to hyperglycemia, and this was not prevented by insulin treatment [19, 20]. TLR4 mediates vascular inflammation and insulin resistance in diet induced obesity. We have previously reported that extracellular HSP60 causes cell apoptosis via TLR4 activation and production of TNF*α* [21]. Chlamydial and other bacterial HSP60 (HSP65), which have high homology to human HSP60, have also been implicated in vascular disease, but because these infectious agents are so commonplace, it has been difficult to irrefutably prove [18, 22, 23]. Cellular (T cells) and humoral (anti-HSP60 antibodies) immune responses potentially also play a fundamental role in triggering the inflammatory process that fuels atherosclerosis [13]. Thus, serum HSP60, which is usually an intracellular protein, can contribute to the inflammatory state seen in diabetes through multiple mechanisms.

 Clinically, elevated HSP60 plasma levels have been found to correlate with increased carotid stiffness in middle-aged individuals [33]. Most significantly, antibodies to HSP60, either mammalian or bacterial (HSP65) were found to correspond to increased intima-media thickness (IMT) in young males (17-18 years.) [34]. These studies support a role for HSP60 and anti-HSP60 antibodies in atherosclerotic disease in the nondiabetic. Correlation may be more difficult to identify in older individuals as a multitude of factors, which increase with age, and contribute to the progression of established atherosclerotic disease. 

### 4.1. HSP60 Antibodies

 A very high titer of HSP60 antibodies was present in our patients, and it did not change with optimization of metabolic control. A number of studies have implicated HSP60 antibodies in endothelial apoptosis and dysfunction [35, 36]. A recent study demonstrated using a randomized double-blind, placebo-controlled cross-over design that simvastatin could lower anti-HSP60 antibody titers [37]. Only 15% of these patients were diabetic, and the anti-inflammatory properties of simvastatin may have been a critical attribute for reduction in anti-HSP60 antibodies [38]. High titers of anti-HSP60 antibody were still present after months of treatment, and these antibodies can interact with HSP60 on the surface of endothelial cells, leading to monocyte recruitment and further inflammation.

### 4.2. Cytokines

 Six different cytokines were measured, and of these only IL-6 was significantly reduced after intensive treatment. This could be secondary to the effect of reduced glucose levels on the inflammatory state of endothelium. However, IL-6 also reflects the low-grade chronic inflammatory state that characterizes diabetes per se, the increased level of insulin resistance, and the increase in visceral adipose tissue, all of them present in the metabolic syndrome [39]. The observed decrease in IL-6 could be due to the reduction of blood glucose as well as of BMI, waist circumference and insulin resistance reached by our patients. This study is one of the few that show this effect in diabetic patients in a context of metabolic syndrome. IL-8 levels were increased, but the decrease in IL-8 with treatment did not reach significance. Other cytokines including TNF*α*, IL-10, and IL-12 did not change with diabetes control. Growing evidence supports a cause-effect relationship between systemic inflammation related to diabetes or obesity, and endothelial inflammation. This is important, as systemic inflammation may make plaques unstable and rupture prone [1–3]. Therefore, any reduction of the systemic inflammation could have a positive effect on atherosclerosis-related disease.

Table 3 compares serum cytokine levels measured at baseline in the current study with recent studies in the literature. These studies covered different populations from different countries. All patients were type-2 diabetics, but the duration of diabetes varied from newly diagnosed to 10 years. Little to no IL-1*β* was detected in any of the studies [24, 26, 27]. IL-6 levels ranged from undetectable in one study to over 38 pg/mL [26–32]. The IL-6 levels for the patients in the current study were in the middle of this range. IL-8 levels in the literature were split with two groups finding levels less than 10 pg/mL, and two other studies, including the current one, finding levels of just over 100 pg/mL [26, 27, 32]. Similarly, IL-10, which was only measured in three studies, was very low in one, 3.78 (current study) and 8.13 pg/mL in the other studies [27, 32]. IL-12 levels were only measured by Mishra et al. with these investigators finding 100 times the levels found in the patients in the current study [28]. TNF*α* levels also had a wide range, but most of the studies, including the current one, detected levels between 0.065 to 6.6 pg/mL, while the remaining 3 had average serum values as high as 50.8 pg/mL [24–29, 31]. Thus, blood cytokine levels vary widely in diabetic populations. 

## 5. Conclusions

 Improved diabetes control was not associated with a decrease in either HSP60 or anti-HSP60, even though IL-6 decreased, suggesting less inflammation. Although diabetes control was significantly improved, as evidenced by a decrease in HOMA, this does not mean that hyperglycemia, which can cause endothelial dysfunction, did not occur intermittently, and that other factors driving inflammation in diabetics were eliminated. Diabetes is a chronic inflammatory disease, and even with good control of blood sugar, there is increased vascular disease. HSP60 and anti-HSP60 antibodies are not reduced by months of improved glucose control and may contribute to the increased incidence of vascular disease in diabetic patients with good glucose control. Finally, IL-6, a metabolic syndrome and atherosclerosis-related inflammatory marker could be of value in clinical practice as an indicator (together with HgbA1c) of the efficacy of the treatment as well as of the efficacy of preventive action on atherosclerosis.

## Figures and Tables

**Table 1:** Characteristics of patients enrolled in the study.

Patient	Age	Sex	BMI		HgbA1C (n.v. 6,6)		HOMA		Waist circum (cm)		Diabetes treatment: drugs (+diet and physical activity)	Lenght of treatment (years/months)	Comorbidties
			Before	After	Before	After	Before	After	Before	After
1	51	M	35	34	8.5	7.2	2.9	3.6	136	112	Gliclazid	0/3	HTN, DL
2	66	M	30	30	15.6	6.0	6.0	6.0	104	100	Repaglinid	0/6	HTN, DL
3	52	F	31	31	7.9	6.3	7.3	6.9	98	89	Metformin	0/6	HTN, DL
4	67	F	36	35	7.3	6.7	5.0	5.2	113	105	Metformin	1/2	HTN, DL, CVA
5	64	M	28	24	7.3	5.6	1.3	0.9	104	93	Metformin	0/6	HTN, DL
6	65	F	27.5	25.5	9.5	6.7	5.1	1.9	113	104	Metformin	1/0	HTN, DL
7	67	M	31	27	7.9	5.8	4.8	2.3	107	96	Metformin	0/5	HTN, DL
8	60	M	31	30	9.4	7.2	5.6	3.9	103	96	Metformin	0/2	HTN, DL
9	74	F	37	33	7.7	6.6	3.2	1.8	124	107	Metformin + Repaglinid	1/2	HTN, DL, Afib
10	67	F	38	34	8.4	6.4	8.7	2.5	136	128	Metformin + Glimepirid	0/6	HTN, DL
11	50	M	29	27	12.0	6.9	3.8	1.6	96	106	Metformin + Glimepirid	0/6	HTN, DL
12	71	F	40	38	8.4	6.5	5.6	3.9	133	127	Metformin	0/8	HTN, DL
13	71	F	43	39	8.2	6.7	5.7	2.6	109	109	Metformin	0/5	HTN, DL
14	73	F	32	29	8	6.9	4.5	3.8	102	102	Metformin	0/8	HTN, DL
15	59	F	41	40	9.1	6.7	10.9	4.0	121	118	Metformin	1/5	HTN, DL
16	55	F	41	39	8.4	6.9	6.0	2.3	123	102	Metformin	0/2	HTN, DL
17	47	M	31	29	8.8	6.6	9.5	3.5	104	96	Metformin	1/9	HTN, DL

Pat: patient; n.v: normal value; circum: circumference; treatment: Treatment; comorbidities: cardiovascular comorbidities; HTN: hypertension; DL: dyslipidemia; CVA: cerebral vascular accident; Afib: atrial fibrillation.

**Table 2:** Significant Pearson correlations for clinical factors and inflammatory markers change with treatment.

Before intensive treatment				
HSP60	IL-10	IL-12	TNF*α*	BMI
−IL-12 0.040	−HSP60 0.051	−HSP60 0.040	+ IL-12 0.014	+ Waist 0.005
−IL-10 0.051	+IL-12 0.030	+IL-10 0.030		
+IL-6 0.055		+TNF*α* 0.014		

After Intensive treatment				
TNF*α*	HOMA	BMI		

+HSP60 0.055	−IL-10 0.009	+Waist 0.010		
+IL-12 0.023				

+ is positive correlation, − negative correlation. Number on right is *P* value.

**Table 3:** Summary of serum cytokine values reported from recent studies.

Reference	IL-1*β* pg/mL	IL-6 pg/mL	IL-8 pg/mL	IL-10 pg/mL	IL-12 pg/mL	TNF*α* pg/mL	Diabetes duration (Years)	Nationality
Mol et al. 1997 [24]	0.816					1.19	10 ± 7^∗^	Dutch
Adachi et al. 2004 [25]						2.4	Unknown	Japanese
Doganay et al. 2002 [26]	<0.05	<0.05	8.3			6.6	6.0 ± 0.6	Turkish
Lee et al. 2008 [27]	0.94	1.47	5.11	0.77		3.95	9.0 ± 5.4	Korean
Mishra et al. 2011^1^ [28]		6.12			118	38.6	newly diagnosed	Indian
Mishra et al. 2011^2^ [28]		9.55			147	50.8	newly diagnosed	Indian
McGee et al. 2011 [29]		7.03				16.9	1 yr. Minimum	British
Park et al. 2011 [30]		1.78					5.1	Korean
Hu et al. 2009^3^ [31]		21.8				0.065	Unknown	Chinese
Hu et al. 2009^4^ [31]		38.5				0.111	Unknown	Chinese
Ozturk et al. 2009 [32]		4.01	104.8	8.13			9.84 ± 7.13	Turkish
Current Study	0	12.05	104.6	3.78	1.41	0.83	6.0 ± 5.3	Italian

Values ± SEM except where indicated. Mishra et al. and Hu et al. both divided their diabetic populations into 2 groups: one by CRP and the other by presence or absence of evidence of atherosclerosis. Last row is baseline values for current study. ^∗^Standard deviation; ^1^CRP< 3, ^2^CRP > 3; ^3^no evidence of atherosclerosis; ^4^evidence of atherosclerosis (increased carotid IMT).
